# Supplementary material for: Development of Low Hysteresis, Linear Weft-Knitted Strain Sensors for Smart Textile Applications
Source: Sensors (Basel). 2022 Oct 10;22(19):7688. doi: 10.3390/s22197688 (PMC9572287; doi:10.3390/s22197688)
Supplement: Supplementary file 1 [file sensors-22-07688-s001.zip › sensors-1903283-supplementary.pdf]

Supporting Information

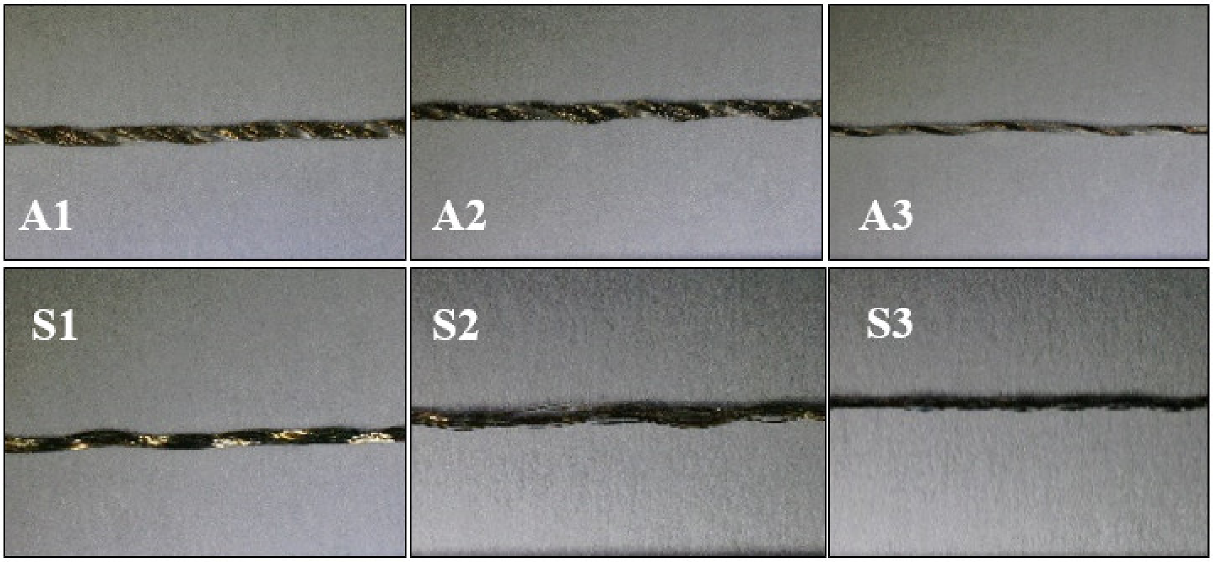

Shieldex (S)				Amann (A)		
**Resistivity (ohm)**	**A1**	**A2**	**A3**	**S1**	**S2**	**S3**
	Tex 28<br>< 530 Ω/m	Tex 62<br><150 Ω/m	Tex 96<br><85 Ω/m	Dtex 235<br>600 Ω/m	Dtex 117<br><1.5 kΩ/m	Dtex 78<br><3.5 kΩ/m

Figure S1. Optical images of conductive yarns in which 'S' refers to the Shieldex yarn (Material: Polyamide 6.6 filament, Metal Plated: 99 % Pure silver) and 'A' refers to Amann yarn (Silver coated Polyamide/Polyester hybrid thread).

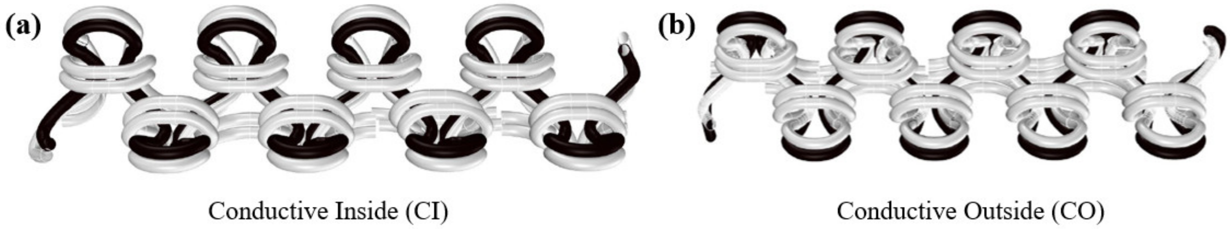

Figure S2. The position of conductive yarn within the co-knit yarn configuration: (**a**) Conductive yarn (black yarn) positioned inside, and (**b**) Elastic yarn (white yarn) positioned inside.

Table S1. The effect of test speed on electromechanical performance of the knitted strain sensors of $NP_9Y_{S1}C_2E_1$, $NP_{10}Y_{S1}C_2E_1$ and $NP_{10}Y_{S1}C_3E_1$.

Samples	Test Speed (mm/min)	Initial Resistance (Ω)	Gauge Factor (GF)	Working Range (%)	Hysteresis
$NP_9Y_{S1}C_2E_1$	30	17.33	1.60	0-22	0.07
	40	17.19	1.80	0-20	0.07
	50	17.22	1.80	0-22	0.07
$NP_{10}Y_{S1}C_2E_1$	30	23.40	1.14	0-23	0.07
	40	23.60	1.11	0-24	0.05
	50	23.03	1.40	0-25	0.07
$NP_{10}Y_{S1}C_3E_1$	30	7.13	0.23	0-36	0.25
	40	7.21	0.37	0-17	0.30
	50	7.21	0.52	0-17	0.27

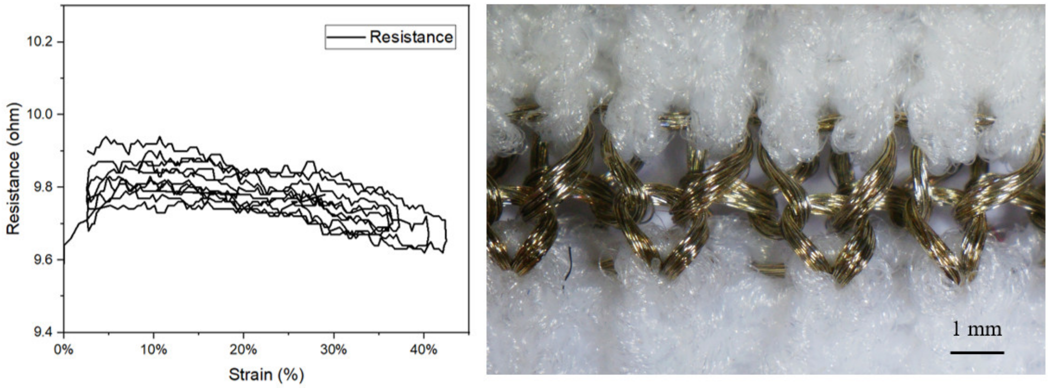

Figure S3. The resistance versus strain graph without E_1 plating/elastic yarn as a co-knit material for the $NP_9Y_{S1}C_2$ sample (**left**)—Optical image of the $NP_9Y_{S1}C_2$ sample without elastic plating yarn (**right**).

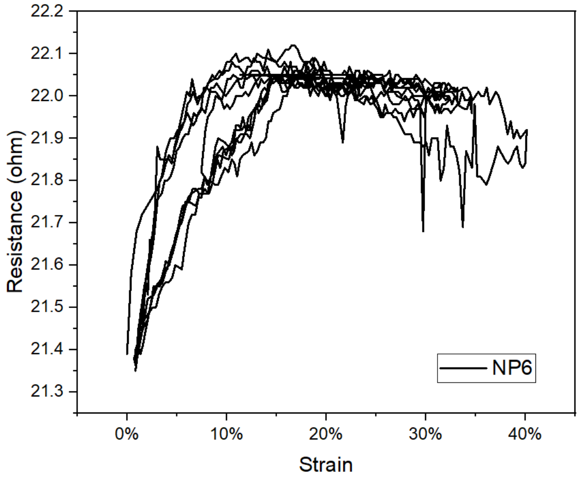

22.2
22.1
22.0
21.9
21.8
21.7
21.6
21.5
21.4
21.3
Resistance (ohm)
0%
10%
20%
30%
40%
Strain
NP6

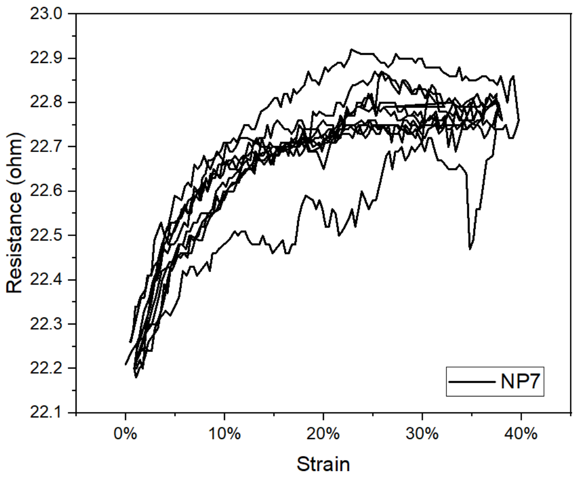

23.0
22.9
22.8
22.7
22.6
22.5
22.4
22.3
22.2
22.1
Resistance (ohm)
0%
10%
20%
30%
40%
Strain
NP7

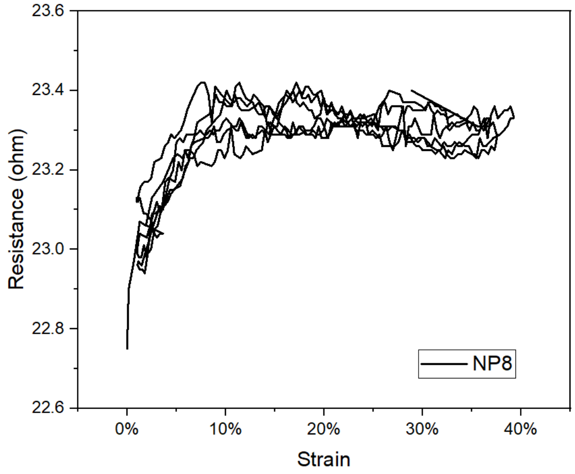

23.6
23.4
23.2
23.0
22.8
22.6
Resistance (ohm)
0%
10%
20%
30%
40%
Strain
NP8

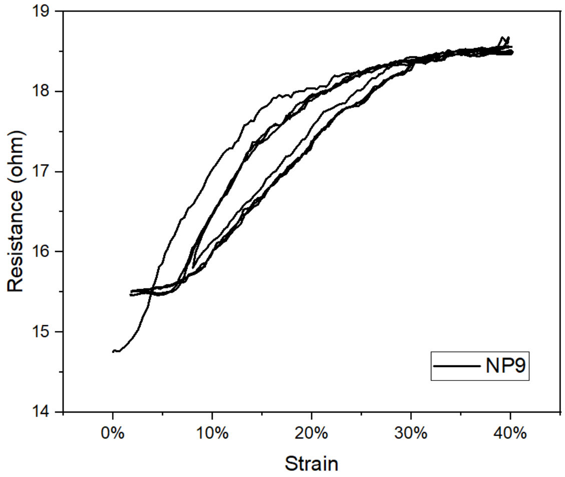

19
18
17
16
15
14
Resistance (ohm)
0%
10%
20%
30%
40%
Strain
NP9

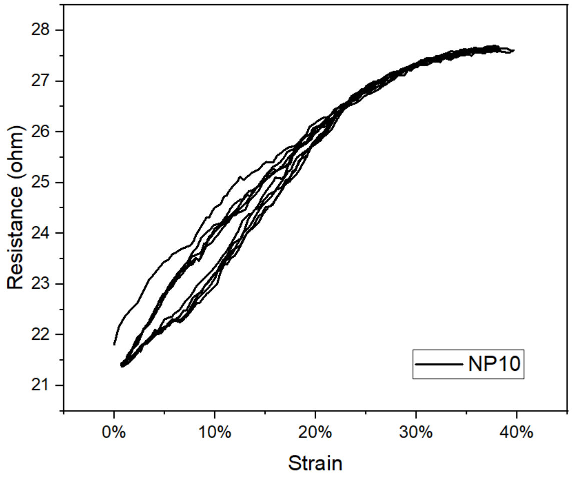

28
27
26
25
24
23
22
21
Resistance (ohm)
0%
10%
20%
30%
40%
Strain
NP10

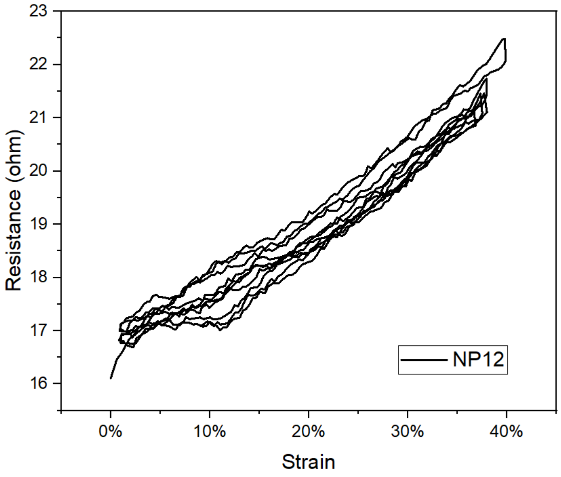

23
22
21
20
19
18
17
16
Resistance (ohm)
0%
10%
20%
30%
40%
Strain
NP12

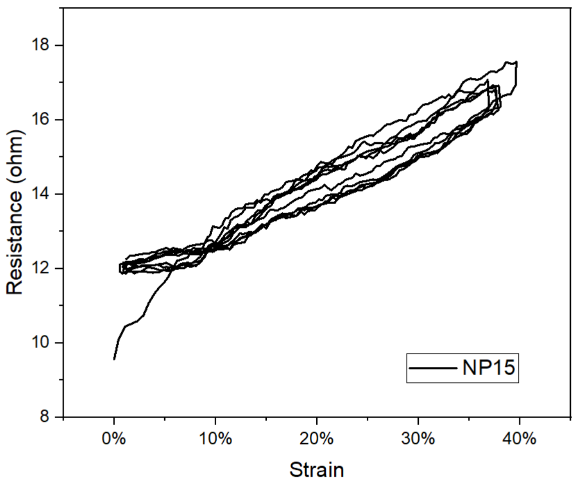

Figure S4. Examples of irregular and regular cyclic patterns: NP$_6$, NP$_7$, NP$_8$, NP$_9$, NP$_{10}$, NP$_{12}$, NP$_{15}$. Resistance versus strain graphs for the series of NP$_x$Y$_{S1}$C$_2$E$_1$.

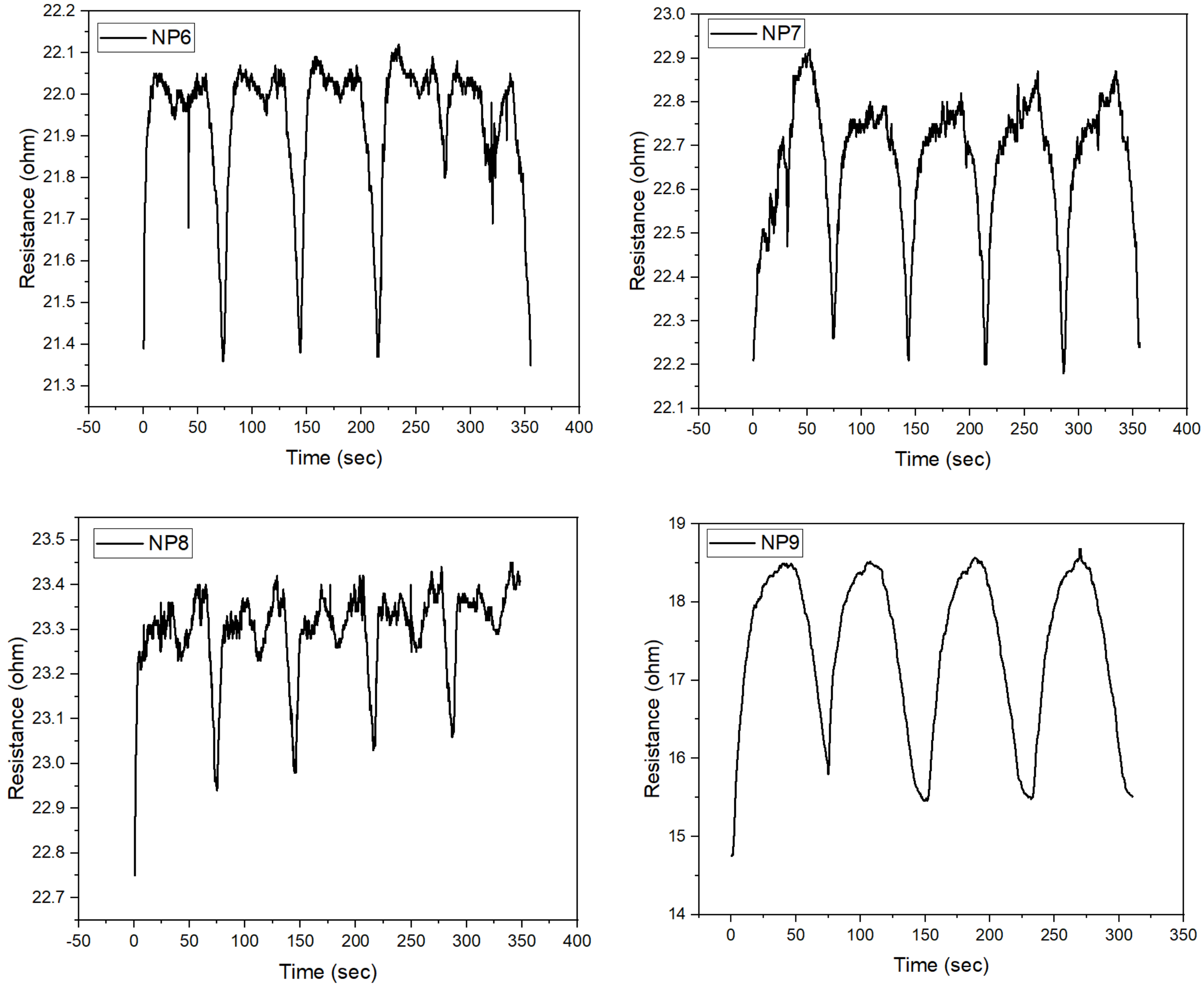

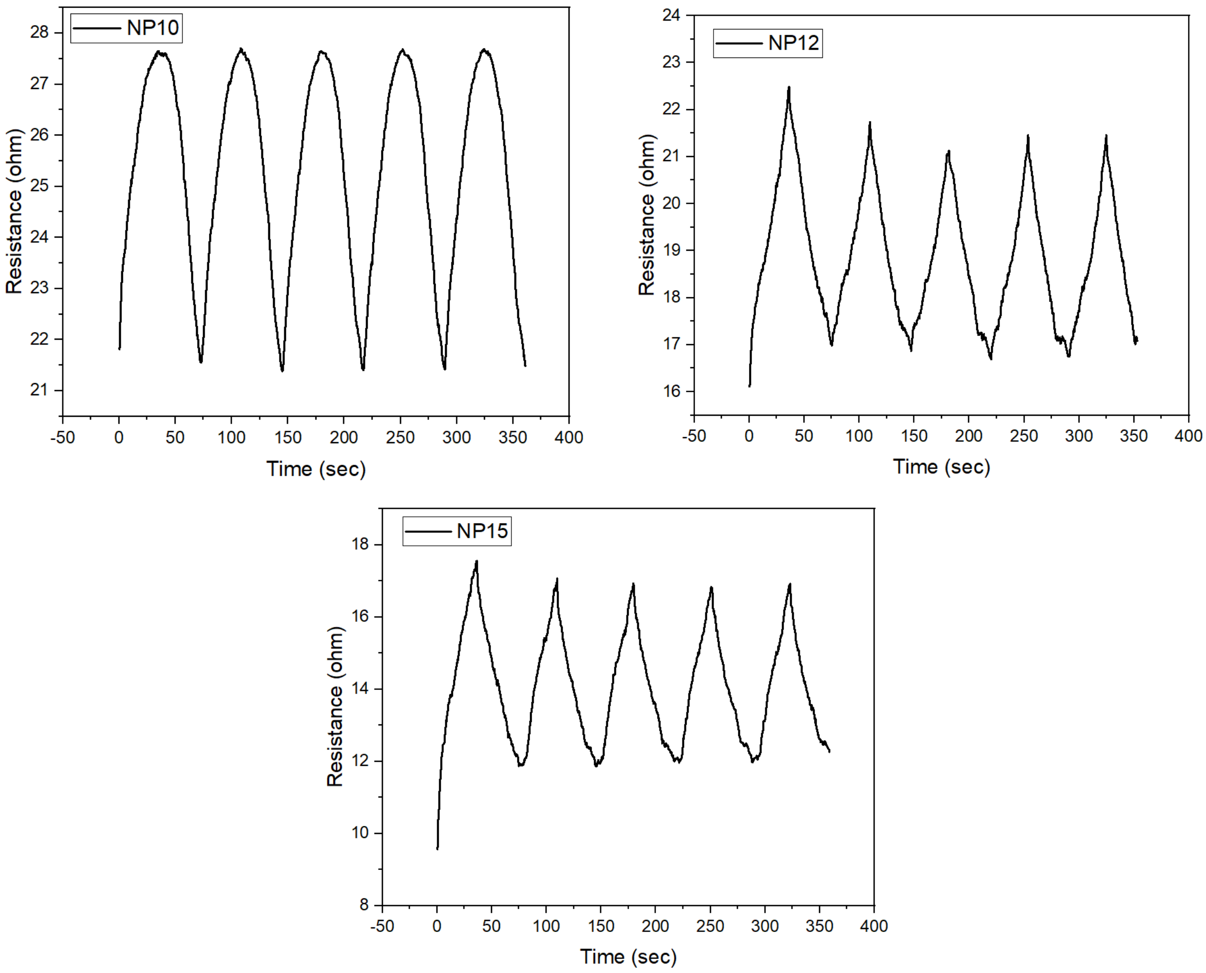

Figure S5. Examples of irregular and regular cyclic patterns: NP_6, NP_7, NP_8, NP_9, NP_{10}, NP_{12}, NP_{15}. Resistance versus time graphs for the series of $NP_xY_{S1}C_2E_1$.

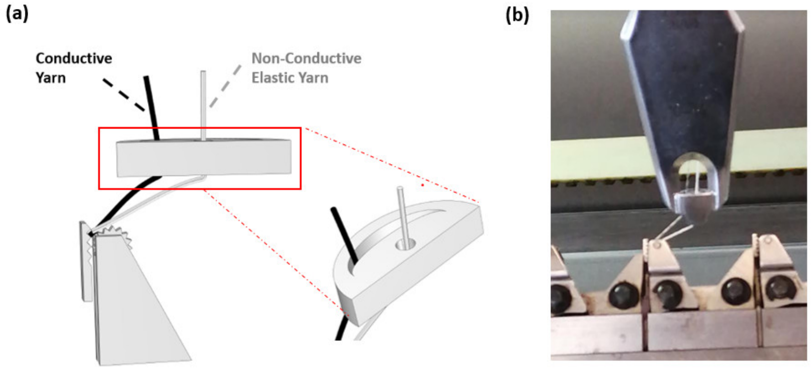

Figure S6. The schematic of yarn carrier (**a**), and picture of plated yarn carrier with 2 yarns consisting of conductive yarn and non-conductive elastic yarn (**b**).

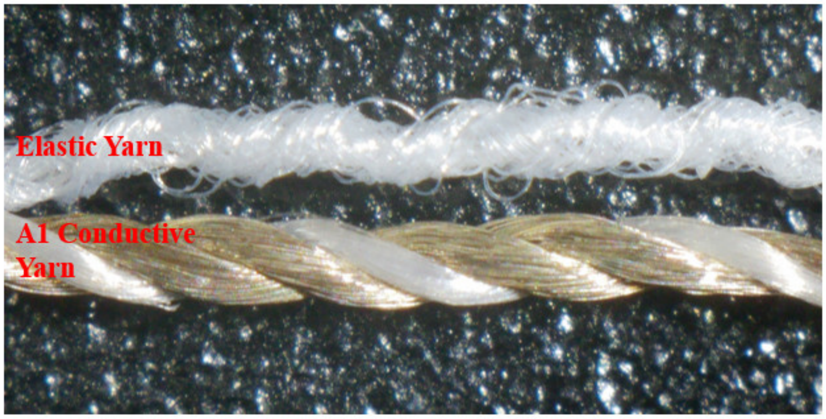

Figure S7. Optical image of plating elastic yarn (white) and A1 conductive yarn (white and silver).
